# Retraction Note: Effects of the TLR4 signaling pathway on apoptosis of neuronal cells in diabetes mellitus complicated with cerebral infarction in a rat model

**DOI:** 10.1038/s41598-025-31863-9

**Published:** 2025-12-15

**Authors:** Chao Li, Li-He Che, Tie-Feng Ji, Lei Shi, Jin-Lu Yu

**Affiliations:** 1 Department of Neurology, The First Hospital of Jilin University, Changchun, 130021 P.R. China; 2 Department of Infectious Diseases, The First Hospital of Jilin University, Changchun, 130021 P.R. China; 3 Department of Radiology, The First Hospital of Jilin University, Changchun, 130021 P.R. China; 4 Department of Neurosurgery, The First Hospital of Jilin University, Changchun, 130021 P.R. China

Retraction of: *Scientific Reports* 10.1038/srep43834, published online 08 March 2017

The Editors have retracted this Article. Following the publication of the Article the Corresponding Author contacted the Journal to request a retraction, stating that the results could not be replicated. Following an investigation, further concerns were raised regarding a number of Figures, specifically:

- Figure 3: a number of panels appear to be identical to panels in Figure 1 of an article by different authors that was simultaneously under consideration at a different journal^1^.
- Figure 3: a number of panels appear to be identical to panels in Figure 1 of an article by overlapping authors that was simultaneously under consideration at a different journal^2^.

The Authors have not responded to requests to provide the original data for this study. The Editors therefore no longer have confidence in the reliability of this Article.

The Authors did not respond to correspondence from the Editors about this retraction.
